# Recent advances in printable thermoelectric devices: materials, printing techniques, and applications

**DOI:** 10.1039/c9ra09801a

**Published:** 2020-02-26

**Authors:** Md Sharafat Hossain, Tianzhi Li, Yang Yu, Jason Yong, Je-Hyeong Bahk, Efstratios Skafidas

**Affiliations:** Department of Electrical and Electronic Engineering, ARC Research Hub for Graphene Enabled Industry Transformation, The University of Melbourne Parkville 3010 Australia mshossain@unimelb.edu.au; Department of Mechanical and Materials Engineering, Department of Electrical Engineering and Computer Science, The University of Cincinnati Cincinnati OH 45221 USA

## Abstract

Thermoelectric devices have great potential as a sustainable energy conversion technology to harvest waste heat and perform spot cooling with high reliability. However, most of the thermoelectric devices use toxic and expensive materials, which limits their application. These materials also require high-temperature fabrication processes, limiting their compatibility with flexible, bio-compatible substrate. Printing electronics is an exciting new technique for fabrication that has enabled a wide array of biocompatible and conformable systems. Being able to print thermoelectric devices allows them to be custom made with much lower cost for their specific application. Significant effort has been directed toward utilizing polymers and other bio-friendly materials for low-cost, lightweight, and flexible thermoelectric devices. Fortunately, many of these materials can be printed using low-temperature printing processes, enabling their fabrication on biocompatible substrates. This review aims to report the recent progress in developing high performance thermoelectric inks for various printing techniques. In addition to the usual thermoelectric performance measures, we also consider the attributes of flexibility and the processing temperatures. Finally, recent advancement of printed device structures is discussed which aims to maximize the temperature difference across the junctions.

## Abstract 2

Additive printing as a low-cost and efficient fabrication technique for thermoelectric device is reviewed targeting the application of energy harvesting from human body.

## Graphical abstract

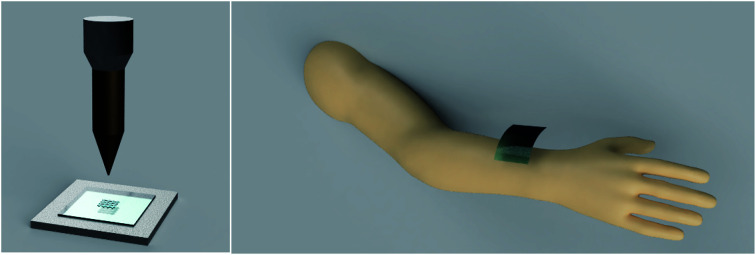

## Introduction

The field of thermoelectrics (TE) has the potential to address two considerable problems in electronic system design. Firstly, thermoelectric generators (TEGs) have the potential to power electronic devices using waste heat and without the need for chemical-based energy storage. Secondly, small-scale thermoelectric coolers can be used to cool down hot-spots in power-dense integrated circuits (ICs). The generated heat density in some high-performance processors can exceed that of a nuclear reactor.^1^ The ability to efficiently extract heat helps maintain the chip performance and increases the reliability of these devices. Wearable electronic devices and sensors especially benefit from TEGs since they can harvest energy from naturally-occurring heat sources such as the human body. However, due to their low-efficiency, toxicity, and high material costs, this field is yet to find broader applicability.

Conventional macro-scale TEGs are made from ingots that are diced typically into mm-sized pallets, which are then electrically connected with metal electrodes and sandwiched between two ceramic plates.^2^ This process involves very high temperatures, expensive equipment and thus not suitable for applications requiring flexible substrates.^3,4^ Moreover, the materials that can be processed using conventional techniques are limited. On the other hand, one of the emerging fabrication methods for flexible devices is additive manufacturing, such as printing.^5^ Researchers have fabricated thin-film transistors,^6^ LEDs,^7^ solar cells,^8^ memristors,^9^ artificial neural networks^10^ using printing technologies. The advantage of printing includes low-temperature vacuum-less process, low-cost equipment, low material wastage, and applicability to flexible substrates.^11^ Considerable research has been directed to improve the printing technology for the mass production of electronic devices.^12^ Some of the persistent challenges are: the low achievable resolution that current printable methodologies can achieve, the rough surface of the substrates, and the non-uniform thickness of the deposited layers of materials, which detrimentally affect the performance of the fabricated transistors.

In contrast to transistors, where the gate length is inversely proportional to maximum switching speed and maximum frequency with a power gain, TEGs do not have as stringent resolution requirements. Most of the TEGs are mm-to-cm scale devices which makes printing an attractive alternative fabrication technique. However, in most cases, the performance of the printed devices are substantially lower compared to their bulk counterparts.

This review focuses on TEGs that have been fabricated using printing technologies. We concentrate on TEGs that operate at room temperature which are suitable for energy harvesting from body heat. We start by introducing the theory behind thermoelectricity and the methodologies that are available to enhance their performance. We review electronic device printing methodologies and present printed TEGs reported in scientific literature and compare their performance. Finally, we discuss various device structures that are utilized to maximize performance for printed TEGs.

## Fundamentals of thermoelectricity

**Fig. 1:**
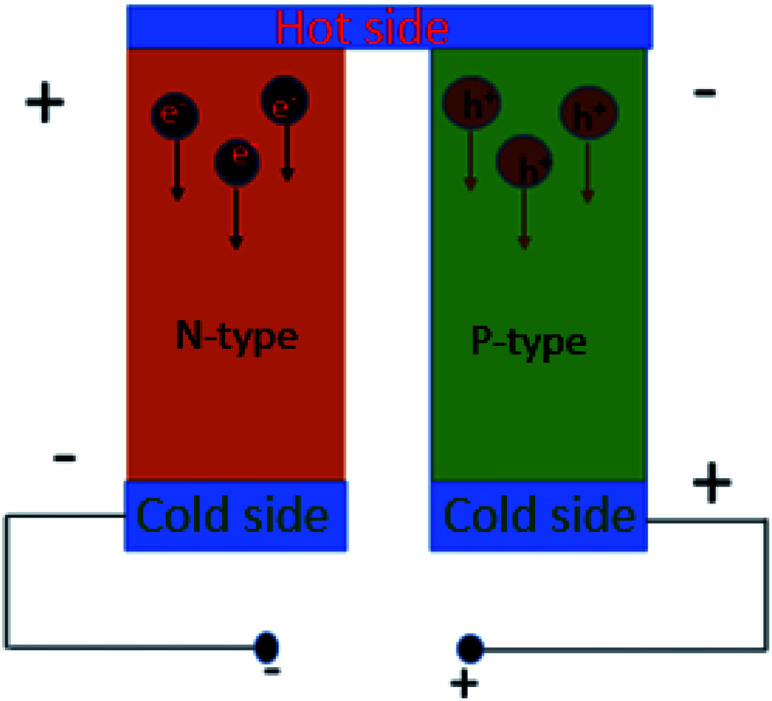
Schematic illustration of the mechanism of voltage generation.

The thermoelectric effects are fundamental solid-state transport phenomena responsible for the direct conversion of thermal energy to electrical energy and *vice versa*. A thermoelectric device generally contains a combination of n-type and p-type semiconductor elements which are connected electrically in series and thermally in parallel. When a temperature gradient is applied across the device, charge carriers at the hot end will acquire increased energy and will diffuse towards the cold end where their energy can be lowered. Opposite potential is generated in n-type and p-type materials where charge carriers are negatively charged electrons and positively charged holes, respectively. So, connecting the p-type and n-type element in series adds up the generated voltage. This is illustrated in Fig. 1 where the voltage between terminal 1 and 2 is the summation of *V*_p_ and *V*_n_. If a load is connected between terminal 1 and 2, an electric current flows, and power is delivered to the load. To evaluate the performance of a thermoelectric device, the most commonly used parameter is the dimensionless figure of merit *ZT*^13^ defined as:1
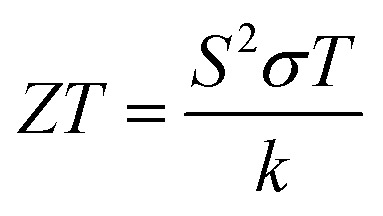
where *T* is the average absolute temperature between the hot and cold sides of the device, *S* is the Seebeck coefficient, *σ* is the electrical conductivity, and *k* is the thermal conductivity. The quantity *S*^2^*σ* is called the power factor, which is determined by the carrier transport. The thermal conductivity consists of two terms, the electrical thermal conductivity *k*_elec_ and the phonon thermal conductivity *k*_ph_, such that *k*_total_ = *k*_elec_ + *k*_ph_. A key area of research effort in thermoelectrics centres around maximizing *ZT*. However, this is challenging as the constituting parameters are interdependent and cannot be optimized individually.^14^

The minimum *ZT* required for a good thermoelectric material will depend on application. If we assume no parasitic heat losses through the gap fillers and sidewalls of TE elements, the maximum achievable output power density can be expressed as:^15^2
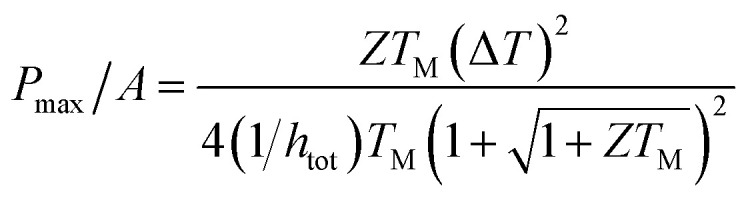
where *h*_tot_ is the total external heat transfer coefficient from both the top and bottom sides, *i.e.*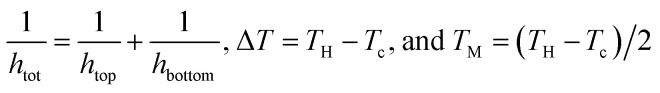.

**Fig. 2:**
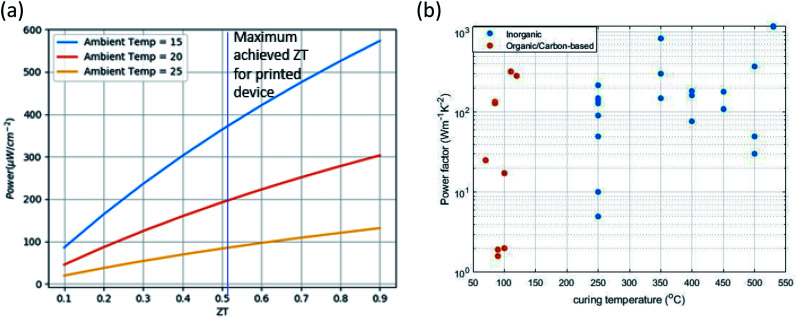
(a) Achievable output power from human body *vs. ZT*. (b) Power factor *vs.* curing temperature for printed TEGs.

If we consider harvesting heat from human body, *h*_top_ will be the heat transfer co-efficient from the TEG to the atmosphere and *h*_bottom_ is the heat transfer co-efficient from TEG to human skin. *T*_c_ is the ambient temperature and *T*_H_ is the temperature of human skin. Assuming *h*_top_ = 10 W m^−2^ K, *h*_bottom_ = 50 W m^−2^ K (ref. 16) and Th = 36 °C, Fig. 2(a) shows the relation between *ZT* and maximum output power density achievable when harvesting heat from the human body. For this application, when the ambient temperature is 20 °C, an output power density of 200 μW cm^−2^ can be achieved with state of the art printed TEG.

## Optimizing thermoelectric materials

Some of the complexity associated with optimizing the components of *ZT* is discussed here. Electrical conductivity is defined by *σ* = *enμ*, where *e* is the charge of electrons, *n* is the carrier concentration and *μ* is the carrier mobility. Carrier mobility can be defined as *μ* = *eτ*/*m*. Here *τ* is mean scattering time of the carrier and *m* is the effective mass. So, conductivity can be increased by increasing the carrier concentration (*n*). However, For metal and degenerate semi-conductors, the Seebeck coefficient drops with increasing carrier concentration. Consequently, optimization of the carrier concentration is critical to obtain the best performance. Another important parameter is the effective charge carrier mass. Higher *m* is related to a flat band near the Fermi surface, which increases the Seebeck coefficient. However, the higher *m* will lower the carrier mobility (*μ*). As mobility is directly proportional to conductivity, lowering carrier mobility will decrease the conductivity. Finally, based on eqn (1), a good thermoelectric material should have high electrical conductivity yet low thermal conductivity. A direct relationship between electronic thermal conductivity (*k*_elec_) and (*σ*) further complicates materials optimization.

There has been significant research effort focussed on optimizing the parameters in eqn (1) to maximize *ZT*. Apart from the well-known thermoelectric material such as Bi_2_Te_3_ or Sb_2_Te_3_, other complex materials including chalcogenides, clathrates, oxides, skutterudites, half-Heusler alloys have been investigated.^4^ However, most of these materials operate efficiently at temperatures much higher than that of the human body. Apart from looking for alternate materials, one of the most successful approaches to improve *ZT* has been to reduce the phonon thermal conductivity without the significant reduction of the power factor.^17,18^ This strategy has been achieved *via* nanostructuring,^19,20^ embedding nanoparticles into semiconductors^21^ or using nanocomposites instead of bulk materials.^22^ The performance improvement is due to the selective scattering of electrons and phonons at interfaces.^23^ Mahan and Sofo investigated the effect of the transmission function of charge carriers on the power factor of thermoelectric materials and suggested that a delta-like transmission function can improve the power factor.^24^ Heremans *et al.*^25^ doped PbTe with Tl, which introduced resonant energy levels within the valence band to create sharp delta-like density of states to improve the power-factor. Another popular method to enhance the Seebeck coefficient is *via* energy filtering. By introducing an energy barrier, low energy electrons can be preferentially scattered while high energy electrons are less affected, thereby enhancing the entropy transport through the material.^21^ Zebarjadi *et al.* proposed the modulation doping scheme to enhance the power factor, where charge carriers are donated from dopants that are confined within the second phase grains to the matrix occupying the majority volume.^26^ Due to the lower scattering in the matrix region void of dopant impurities, the electrical conductivity of the two-phase composite was higher than that of the conventionally doped matrix, resulting in an enhanced power factor. Finally, Boukai *et al.*^18^ proposed and implemented the possibility of utilizing the phonon drag effect^27–29^ to enhance the Seebeck coefficient at low temperatures. Recently it has been showed that phonon drag could prevail at a higher temperature for lower dimensional materials like silicon nanowires resulting in a very high *ZT*.^18^

When it comes to the printing of TE devices, precursor materials need to be converted to functional inks that are printable. This adds an extra optimization step for printed TEGs. To date, most of the printed room temperature inorganic TEGs have used Bi_2_Te_3_, Sb_2_Te_3_ and their alloys. Other inorganic materials used in printed TE include Bi_2_Te_*x*_Se_3−*x*_, Ca_3_Co_4_O_9_ and ZnO doped In_2_O_3_.^30^ There are two common approaches to prepare functional inks using these materials. One includes ball milling the inorganic materials to nanoparticle forms, then dispersing them in aqueous solutions or polymer matrices. After printing the ink, a curing step is needed to get rid of the solvent or polymer. Another method is to use precursors of the functional materials as inks. Once printed, a reaction step is needed on the substrate to convert the precursor to the desired material. A popular example of this method is the sol–gel process to print metal oxides.^31^ Precursor solution of metal salts is converted to gel *via* hydrolysis and printed on a substrate. Post-processing like sintering converts the gel to a thin film of metal oxide. In both methods, the quality of the printed film depends on how densely packed the nano-particles are in the final film. This is directly related to the curing/sintering step. Typically, the higher the temperature is used for the curing/sintering, the less solvent/polymer remains in the film. Also, higher temperatures densify the film. Unfortunately, most of the flexible substrates used cannot withstand high temperatures. So the range of substrate that can be used becomes limited. This makes the curing temperature another optimization parameter. Recently, photonic sintering has been proposed, where UV light is used to sinter the ink without additional heat treatments.^32^

Apart from the inorganic materials, several organic polymers have been investigated for thermoelectric applications.^33–36^ One advantage of organic polymers in printing is low curing temperature.^37,38^ This enables them to be printed in a wide range of substrates. However, the thermoelectric parameters, conductivity and Seebeck co-efficient, of organic polymers is lower than established in-organic materials. Consequently, several unique approaches have been proposed by the research community to enhance the thermoelectric performance of organic materials.^39–41^

Both conducting and non-conducting polymers are utilized to fabricate thermoelectric devices. Non-conducting polymers are generally used as matrices, where thermoelectric nanoparticles are added as the active material. Apart from the thermoelectric performance of the nanoparticles, the ink performance will depend on how the nanoparticles are dispersed within the polymer matrix. For instance, Prabhakar *et al.* dispersed carbon nanotubes (CNTs) in insulating polydimethylsiloxane (PDMS) elastomer to achieve a thick and flexible thermoelectric film.^39^ The thermally insulating elastomer reduced the thermal conductivity, and a high power factor was achieved through the tunneling transport in the CNT networks. For conductive polymers, the performance depends upon the doping level, morphology of the chain and the chemical structure of the monomers. Bubnova *et al.*^40^ reduced the doping of poly(3,4-ethylenedioxythiophene)-tosylate (PEDOT:Tos) by exposing it to reduction agents. By optimizing the doping, they were able to increase the power factor by one order of magnitude. Adding inorganic nano-structures in conductive polymers is another approach to improve the performance. For example, Meng *et al.* were able to improve the power factor of polyaniline (PANI) by adding CNTs and graphene flakes.^42^ See *et al.* utilized the high Seebeck co-efficient of Te-nanorods and the low thermal conductivity of PEDOT:polystyrene sulfonate (PSS) by mixing them into a nanocomposite.^43^ Other research has shown that using longer chains of PEDOT, both the Seebeck coefficient and conductivity can be increased.^44^ Since the transport in a conductive polymer is dominated by thermally activated electron, hopping between inter-chain and intra-chain sites, the transport heavily depends on the morphology of the polymer.^45^ Kim *et al.* used ethylene glycol (EG) to remove the excess insulating PSS from PEDOT chains. They achieved a power factor as high as 469 μW m^−1^ K^−2^.^41^ Since most of these polymers are synthesized in aqueous solution, they can be directly printed without any further ink processing, provided the solution was sufficiently viscous. Also, lower temperature is required in the curing step to get rid of the solvent. The relation between curing temperature and power factor found in literature is shown in Fig. 2(b). We can see a trend of better performance for higher curing temparature for inorganic materials. On the other hand, organic materials require much lower curing temperature.

## Printing methods

**Fig. 3:**
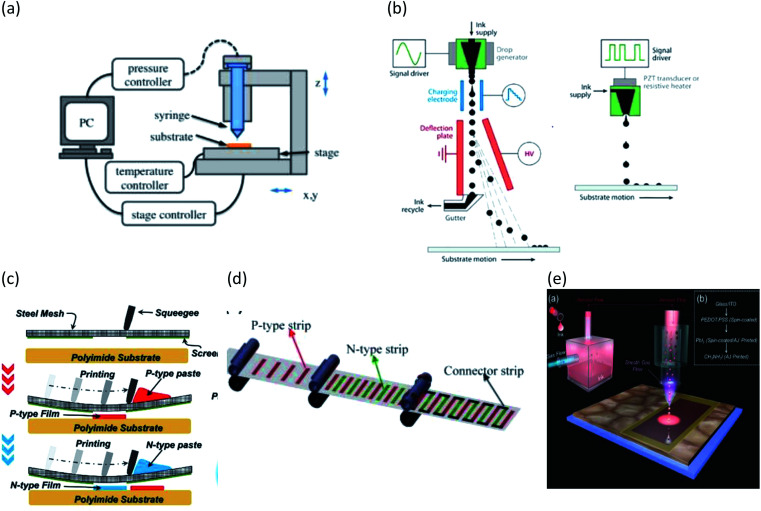
Schematic diagram of different printing method (a) dispenser printing (adapted with permission fromref. 51). (b) Continuous and drop-on-demand inkjet printing. (c) Screen printing (adapted with permission from ref. 108). (d) Roll-to-roll printing (adapted with permission from ref. 101) and (e) aerosol jet printing (adapted with permission from ref. 90).

In this section, we introduce various state-of-the-art printing techniques used for electronic and thermoelectric devices and discuss their advantages and disadvantages. The schematic of these printing methods are illustrated in Fig. 3.

### Dispenser printing

In dispenser printing, a nozzle dispenses continuous filament of ink (*via* pneumatic pressure) slurries on a substrate. An x–y stage, generally controlled by a computer, moves the substrate to achieve the desired design topology. By controlling the ink rheology, stage movement speed, and the distance between nozzle and substrate, conformal writing with feature size down to 250 nm can be achieved.^46,47^ Moreover, dispenser printing is capable of printing very thick films up to 200 μm.^48^

Recently, electric field-based dispensing has been proposed, instead of pneumatic pressure based dispensing. The electrohydrodynamic (EHD) deposition technique relies on applying an electric field to create the force to propel ink from the nozzle onto the target substrate. By using high-frequency pulses for the EHD system, a high spatial resolution down to 100 nm was achieved.^49^ Using this technique, researchers have printed thin-film transistors,^50^ light-emitting diodes,^51^ memristors,^9^ artificial neural networks^10^ and QLEDs.^52^

The main advantage of dispenser printing is low material wastage and mask free patterning. However, preparing the highly concentrated and viscous ink is challenging.

One of the first reported printed TEGs was dispenser printed n-type Bi_2_Te_3_ and p-type Sb_2_Te_3_.^48,53,54^ A polymer binder was used to make pastes, which was then dispenser printed on a polyimide substrate. Due to the lower temperature tolerance of the polyimide substrate, the device was cured at a relatively low temperature, 250 °C. The device performance was two orders of magnitude lower than bulk due to the presence of polymer binder in the system, which reduces the electrical conductivity. The same group later explored several techniques to improve the performance, including mechanical alloying and Se and Te doping.^55,56^ They also integrated their dispenser printed devices with disperser printed micro-batteries demonstrating a complete power supply for a wearable sensor network.^57^ Wu *et al.* combined selective laser melting (SLM) with dispenser printing to achieve device performance comparable to bulk manufacturing technique.^58^ However, using SLM complicates the fabrication process significantly. The dispenser printing method was also utilized to fabricated cross-plane TEGs. Jo *et al.* utilized thick PDMS film with holes as mold.^34^ p-Type and n-type Bi_2_Te_3_ mixed with a polymer binder, was dispenser printed to fill up the holes. The device was annealed at 100 °C. Their 50 × 50 mm device was able to generate 2.1 μW power at a 19 K temperature difference (Table 1).

**Table 1:** Dispenser printed TEGs

p-Type	n-Type	Max temp. (°C)	Substrate	Performance (μW m^−1^ K^−2^)	Ref.
Sb_2_Te_3_-epoxy resin composite	Bi_2_Te_3_-epoxy resin composite	250	Polymide subsrate	p-Type: 150	48
				n-Type: 130	
Sb_2_Te_3_-epoxy composite	Bi_2_Te_3_-epoxy composite	350	Glass	p-Type: 840	53
				n-Type: 150	
—	Mechanical alloyed Bi_2_Te_3_ (1% Se)	350	Flex-PCB	n-Type: 300	56
Bi_0.5_Sb_1.5_Te_3_ with 8 wt% extra Te-epoxy	Bi-epoxy composite	250	—	n-Type: 90	59
				p-Type: 5	
—	Bi_2_Te_2.7_Se_0.3_	Selective laser melting	—	n-Type: 1500	58
p-Type Bi_2_Te_3_	n-Type Bi_2_Te_3_	100	PDMS thick film	—	34

### Inkjet printing

Inkjet printing is a derivative of dispenser printing where ink drops exit the nozzle by a dynamic process. Inkjet printing is generally categorized into the continuous inkjet and the drop-on-demand (DoD) method. Due to its material relevance for thermoelectric device fabrication, we will primarily focus on DoD printing. By controlling the contraction expansion of the piezoelectric actuator, individual ink droplets can be jetted out from the nozzle forming the desired pattern on the substrate. The printed pattern can be easily modified by changing the digital file that controls the actuator. The printability depends on the nozzle radius and viscosity, density and surface tension of the ink.^60^

Inkjet printing has been intensely investigated as a low cost fabrication technique for the electronics industry. Chen *et al.* have demonstrated fully inkjet-printed transistors with mobility of approximately 1 cm^2^ V^−1^ s^−1^ and an on-to-off ratio of ∼10^7^.^6^ Liu *et al.* demonstrated inkjet printed QLEDs reaching the maximum luminance of 89 500 cd cm^−2^.^61^ Furthermore, researchers have investigated the viability of inkjet-printed image sensors.^62^

In spite of the wide application, commercial products using inkjet printed materials are not currently widely available. This can be attributed to the following: (1) when the size of the particles contained in ink is in the order of nozzle diameter, the printer nozzle gets easily clogged, affecting the printing pattern. Since printing resolution depends on the nozzle size, using a larger nozzle to avoiding clogging affects the achievable resolution. (2) Droplets form a ‘coffee ring’ on the substrate caused by non-uniform drying of the ink. The centre of the coffee ring is thinner than the edge leading to non-uniformity and undesirable device characteristics. There have been several efforts to minimize non-uniform printing, including electrowetting,^63^ eddies,^64^ and particle trapping.^65^ However, these methods have not been thoroughly investigated in the context of ink-jet printing.

Inkjet printing is another highly used method to print thermoelectric devices (Table 2). Since the films are thin, most of the inkjet printed TEGs are planar device. Both inorganic, polymer and carbon based materials have been printed using ink-jet printed techniques. One of the first reported inkjet printed inorganic TEGs were fabricated using p-type Sb_1.5_Bi_0.5_Te_3_ nanoparticles and n-type Bi_2_Te_2.7_Se_0.3_ nanoparticles based inks.^66^ Commercial stabilizer was used to formulate aqueous solutions of the nanoparticles and aco-solvent was used to achieve the viscosity and surface tension suitable for inkjet printing. The Ag nanoparticle-based electrodes were also inkjet printed making the device fully printed. The group was also able to print the TEGs on flexible polyamide substrate. They could achieve a power factor of 77 μW m^−1^ K^−2^ for n-type and 183 μW m^−1^ K^−2^ for p-type materials. Unfortunately, the process requires a high annealing temperature of 400 °C, necessary to decompose the polymeric stabilizer making it unsuitable for many flexible substrates.

**Table 2:** List of inkjet printed TEGs

p-Type	n-Type	Max temp.	Substrate	Performance (μW m^−1^ K^−2^)	Ref.
Sb_1.5_Bi_0.5_Te_3_	Bi_2_Te_2.7_Se_0.3_	400 °C	Glass/polymide	p-Type: 183	66
				n-Type: 77	
	Bi_2_Te_3_ nanowires	400 °C	Glass	n-Type: 163	67
(Bi_0.5_Sb_1.5_Te_3_) nanowires	(Bi_2_Te_3_) nanowires	450 °C	Polymide	p-Type: 180	68
				n-Type: 110	
(PEDOT:PSS-ink), ZnO-ink	—	150 °C	Glass	—	70
PAA doped carbon nanotube (CNT)	PEI doped carbon nanotube (CNT)	85 °C	Flexible cable	p-Type: 129	37
				n-Type: 135	
PEDOT:PSS	(PEDOT)_*x*_V_2_O_5_	100 °C	Photo-paper	p-Type: 17.12	38
				n-Type: 2.0	
PEDOT:Tos	—	110 °C	Silicon substrate	p-Type: 324	40
(Poly[Cu_*x*_(Cu-ett)])/PVDF	Poly[K_*x*_(Ni-ett)]/PVDF	90 °C	PET	p-Type: 1.92	71
				n-Type: 1.58	

Chen *et al.* was able to improve the performance of n-type material utilizing nano-structuring effect. Their Bi_2_Te_3_ nanowires based n-type ink could achieve a power factor of 163 μW m^−1^ K^−2^ at room temperature.^67^ The same group recently used nanowires for both n-type and p-type material as well as printing them on flexible polymide substrates.^68^ Their n-type and p-type ink consisted of BiTe and Bi_0.5_Sb_1.5_Te_3_ nanowires and a eutectic gallium–indium (EGaIn) liquid metal as the electrode. Even though they did not use any stabilizer, a high annealing temperature of 450 °C was required to sinter the ink.

One solution to high annealing temperature is to use polymer-based organic thermoelectric material. By controlling the oxidation of PEDOT:Tos, Bubnova *et al.* were able to achieve much higher power factor compared to many printed inorganic TEGs using annealing temperature of only 110 °C.^69^ Since PEDOT:Tos in not soluble, a solution of EDOT monomer and oxidant (Fe(Tos)_3_) were inkjet printed on a warm Ag electrode to facilitate *in situ* polymerization to fabricate the TE leg. Other reports on PEDOT based inkjet printed device include mixing with inorganic nanoparticles. Besganz *et al.* mixed ZnO nanoparticle with PEDOT:PSS-ink and varied its concentration.^70^ The results showed mixing 20% ZnO improves the performance compared to pure PEDOT:PSS, but adding more ZnO degrades the performance. A lower annealing temperature of 150 °C was also used, which might be too low for the activation of ZnO.

Most of the conductive polymers are p-type material. To this end, Ferhat *et al.* inkjet printed composite of PEDOT and V_2_O_5_, 5H_2_O gel that performed as the n-type component of the TEG.^38^ Detergent additives (Triton X-100) were used to control the viscosity of the ink. PEDOT:PSS, (PEDOT)_*x*_V_2_O_5_ and silver ink was printed on photo paper to constitute the n-type leg, p-type leg and electrodes. Because the device was all polymer based, an annealing temperature of 100 °C was sufficient to cure the device. Here, the power factor of the n-type leg was two orders of magnitude lower compared to other printed inorganic materials. Moreover, the PEDOT:PSS was not optimized resulting in significantly lower power factor for p-type leg.

On a different front, carbon-based inks showed promising performance as inkjet printed devices. Park *et al.* used CNT as both p-type and n-type legs by doping with PAA and PEI respectively.^37^ They controlled the doping to obtain optimal carrier concentration and achieved power factor of 129 and 135 for p-type and n-type legs. This is comparable to inkjet printed inorganic device making them a promising candidate for fully printed TEGs. The annealing temperature of the ink was 85 °C even though the electrodes were cured at 120 °C.

Jiao *et al.* inkjet printed insoluble and infusible metal coordination polymers.^71^ Both n (poly[K_*x*_(Ni-ett)]/PVDF)- and p-type (poly[Cu_*x*_(Cu-ett)]/PVDF) composites were obtained by ball-milling. The composite material maintained the performance of bulk reaching PF of 1.92 and 1.58 μW m^−1^ K^−2^ at 400 K for n- and p-type composite respectively.

### Screen printing

Screen printing is a popular technique that has been widely used in commercial printing processes. Recently, this technique has found popularity in electronics applications.^72^ Typically, stencils or patterned mesh are used as the template. Viscous ink is forced through the mesh using a squeegee type device. The deposition resolution and thickness of the pattern depends on the viscosity of the ink used and the density of the mesh. A 100 μm thick layer with a 100 μm resolution can be achieved with an ink viscosity of 50 pascal.^73^ Researchers have demonstrated mass production capabilities of photovoltaic cells,^74^ RF antennas^75^ and light emitting diodes^76^ using the screen printing method.

The advantage of screen printing, as compared to other printing methods, include the broader range of available substrates and inks that are compatible with this method. The disadvantage of this method includes high viscosity requirement of the ink and the relative longer drying time. Moreover, the screen printed film is known to have a rough surface and are susceptible to cracking and delamination.^74,77^

Screen printing is one of the most popular methods used to print thermoelectric devices (Table 3). Both organic and inorganic inks has been used in conjunction with this method. The first reported screen printed TEG contained p-type Sb and n-type Bi_0.85_Sb_0.15_.^78^ The authors investigated different binders including ethylene glycol, 2-component epoxy glue and PMMA, and could achieve a power factor of 90 μW K^−2^ m^−1^. Kapton tape was used as the substrate and were able to coil up the devices, demonstrating their mechanical flexibility. However, none of the curing conditions were described. Research effort^79–85^ has improved the performance of BiTe and BiSb based TEGs and lowered the curing temperature by optimizing the ink, using suitable binder additive, mechanical alloying, and doping. Curing temperature as low as 250 °C has been able to achieve power factors of 215 μW m^−1^ K^−2^ and 141 μW m^−1^ K^−2^ for p-type Sb_2_Te_3_ and n-type Bi_3.2_Sb_1.8_.^84^ Kim *et al.* proposed a novel substrate-less TEG by screen printing Bi_2_Te_3_ and Sb_2_Te_3_ paste on glass fabric.^86^ Since this method does not use any substrate, a high curing temperature of 530 °C was used. As a result they could achieve higher power factors of 1066 μW m^−1^ K^−2^ for n-type and 1166 μW m^−1^ K^−2^ for p-type material.

**Table 3:** Screen printed TEGs

p-Type	n-Type	Max temp.	Substrate	Performance (μW m^−1^ K^−2^)	Ref.
Sb	Bi_0.85_Sb_0.15_	—	Kapton	p-Type: 10	78
				n-Type: 90	
Bi_0.5_Sb_1.5_Te_3_	Bi_2_Sb_0.3_Te_2.7_	250 °C	Al_2_O_3_	p-Type: 10	79
				n-Type: 50	
	Bi_2_Te_3_	500 °C	SiO_2_/Si	n-Type: 30	82
ZnSb	CoSb_3_	500 °C		p-Type: 370	85
				n-Type: 50	
Sb_2_Te_3_	Bi_3.2_Sb_1.8_	250 °C	Kapton	p-Type: 215	84
				n-Type: 141	
Silver	Nickel	350 °C	Polyimide		
PEDOT:PSS + 5% ethylene glycol	—	70 °C	Paper	p-Type: 25	87
CNT–polystyrene composite	—	—	Polyethylene naphthalate film	p-Type: 0.15	88
Ca_3_Co_4_O_9_	(ZnO)_5_In_2_O_3_	1400 °C	Alumina	p-Type: 1.6	30
				n-Type: 1.4	
Sb_2_Te_3_	Bi_2_Te_3_	530 °C	Glass fabric	p-Type: 1200	86
				n-Type: 1175	

Apart from BiTe and BiSb based TEGs, Rudež *et al.* screen printed Ca_3_Co_4_O_9_ as p-type leg and a (ZnO)_5_In_2_O_3_ as n-type leg to print TEGs on alumina substrate.^30^ They also screen printed the electrodes making it a fully printed TEG device. However, because of the ink formulation, the process required very high temperatures of up to 1400 °C.

Similar to other printing techniques, by using organic and carbon-based material lower curing temperature can be achieved. Wei *et al.* demonstrated screen printing process to print PEDOT:PSS on paper.^87^ Their inks consisted of aqueous PEDOT:PSS with 5% ethylene glycol. Their curing temperature was 150 °C. Their paper substrate was thermally stable compared to other polymer based substrate approaches. Moreover, their aqueous solution had better wettability on paper substrates. Suemori *et al.* used ink composed of CNT and dissolved polystyrene in 1,2-dichlorobenzene to screen print on polyethylene naphthalate substrate.^88^ However, the process is not fully printable since vacuum deposition was used to form the electrodes.

### Aerosol jet and spray printing

Aerosol jet printing is another popular method for printable electronics. In this method, an atomizer aerosolizes the ink into liquid particles of size ranging from 20 nm to 5 μm. Inert gas or compressed airflow is used to transfer the ink particle to the substrate. Several electronic devices, including thin film transistor displays^89^ and solar cells,^90^ have been printed using aerosol jet/spray printing.

The main advantage of spray printing is the higher allowable distance between print head and substrate making it possible to print on non-flat and non-smooth substrates. The achievable resolution is higher than inkjet printing.^91^ However, edge sharpness in lower and localized crystallization caused by the sheath gas can detrimentally affect the bonding layer quality (Table 4).

**Table 4:** Aerosol jet/spray printed TEGs

p-Type	n-Type	Max temp.	Substrate	Performance (μW m^−1^ K^−2^)	Ref.
H_2_SO_4_ treated tellurium-PEDOT:PSS hybrid composite		120 °C	Glass or flexible polyimide	p-Type: 284	92
CNT doped P3HT			Polymide	p-Type: 325	93
Sb_2_Te_3_ + MWCNT + PEDOT:PSS				p-Type: 41	94
BiTe_2.7_Se_0.3_ nanoplate		Photonic sinteritng		p-Type: 730	95

Canlin *et al.*^94^ used an aerosol-jet printing technique to fabricate TEG on flexible substrates. The unique ink consisted of well-dispersed high-S Sb_2_Te_3_ nanoflakes, high-conductive multi-walled carbon nanotubes (MWCNTs) and PEDOT:PSS. Using optimal composition and surface treatment, power factors of ∼41 μW m^−1^ K^−2^, were achieved. Recently, Mortaza *et al.*^95^ achieved a record high power factor of 730 μW m^−1^ K^−2^ for Bi_2_Te_2.7_Se_0.3_ nanoplate based inks using a combination of aerosol jet printing and photonic sintering. Cheon *et al.* used spray painting techniques to print the nanocomposite of CNT and P3HT.^93^ They used a nozzle diameter of 200 μm and polyamide was used as the substrate. The p-type composite was able to produce a power factor of 325 ± 101 μW m^−1^ K^−2^. Base *et al.* treated tellurium-PEDOT:PSS composite with H_2_SO_4_ and spray printed them. By optimizing the H_2_SO_4_ treatment, they achieved a power factor of 284 μW m^−1^ K^−2^. Till now, only p-type material has been aereosol jet printed, leaving room for further research on n-type material.

### Roll-to-roll printing

Roll-to-roll printing is similar to the screen printing technique, where a rotary screen is used for continuous processing of the web (substrate). Hard contact compression is used to transfer the ink to the substrate. This is a high resolution and high throughput process and has been widely used to fabricate both active and passive electronic devices.^96^ Dilfer *et al.* demonstrated a fully R2R printed metal oxide thin film transistor.^97^ Moreover, R2R printing has been used to fabricate solar cells^8^ and light emitting diode.^7^

The main advantage of R2R printing is the high throughput, making it the most lucrative printing technique for high volume production. However, making a multilayer device is challenging.

Even though roll to roll printing method is becoming increasingly popular for applications like solar cell, and transistors, it is not yet widely investigated for TEGs (Table 5). Søndergaard *et al.* used PEDOT:PSS and silver ink to print 18 000 serially connected TEG legs.^100^ The printing was undertaken on PET foil in a three step process that included printing the bottom electrode, PEDOT:PSS and the top electrode. They achieved a speed of 300 junctions per minute, demonstrating mass producibility of these TEGs. However, the measured Seebeck co-efficient of their devices was much lower than other reported values. This difference can be attributed to the their unoptimized PEDOT:PSS solution.

**Table 5:** Brush painted and roll-to-roll printed TEGs

	p-Type	n-Type	Max temp.	Substrate	Performance (μW m^−1^ K^−2^)	Ref.
Brush paint	BiSbTe	Sb_2_Te_3_-based chalcogenidometalate (ChaM) for n-type BiTeSe	—	Polyimide	—	98
	—	Bi_2_Te_2.7_Se_0.3_	400 °C		—	99
Roll-to-roll	PEDOT:PSS		140 °C	PET foil	—	100
	PEDOT:PSS	n-Doped graphene		Plasma treated plastic	—	101
	SWCNT/PEDOT:PSS	SWCNT/PVP	Infrared heating	Polyimide	p-Type: 0.02	102
					n-Type: 0.24	

Zhang *et al.* used roll to roll printing to print both p-type and n-type legs.^101^ PEDOT:PSS mixed with DMSO was used as the p-type ink and n-doped graphene was used as the n-type ink. They used three sequential rollers to transfer the complete pattern simultaneously. Their printing speed was 15–20 mm min^−1^. Their research monograph did not characterize the performance of individual legs of the TEGs but reported an overall output power of 0.24 mW m^−2^ at a temperature of 10 °C. Single wall carbon nanotubes were also used as inks in roll-to-roll printing.^102^ They used infrared heating to sinter the ink. In this process only the inks can be selectively heated without heating up the substrate. This method can enable the usage of substrates that cannot withstand high temperature sintering.

To our knowledge, no inorganic material has been used in roll-to-roll printed TEGs. Moreover, the performance in the roll-to-roll printed TEGs are significantly lower than TEGs reported by other printing technique using the same material. This opens up a significant research opportunity in this field.

### Brush printing

Brush painting is a simple and cheap method which requires a template and brush to print pattern on a substrate.^99^ The thickness of the film depends on parameters such as the viscosity of the ink and the brush speed. This method is desirable for proof of concept experiments since it doesn't require expensive equipment or setup, but it is limited by low resolution and speed. Moreover, a template is required which requires an extra fabrication step.

Park *et al.* proposed brush printing to print on surface of any geometrical shape.^98^ Their brush printing method utilized Bi_2_Te_3_ based organic ink which used Sb_2_Te_3_ chalcogenidometalate as a sintering aid. Using this printing method, the researchers were able to print directly on hemisphere surface. Another reported TEG using brush printing method utilized custom designed Bi_2_Te_2.7_Se_0.3_ based ink. The films were 100–150 μm thick making the device suitable for through plane device (Table 6).^99^

**Table 6:** Comparison among different printing methods

Method	Details	Speed	Ink compatibility	Printed structure
Dispenser printing	-Stage needs to be moved based on the pattern	Slow	-Compatible for both organic and inorganic inks	-Only prints on flat surface
	-Mask free method		-Larger particle size can block nozzle	-Multiple run needed to print vertical structure
	-Needs preussre or electric field for ink dispension			-Small feature size can be achieved
Inkjet printing	-Mask free method	Slow	-Compatible for both organic and inorganic inks	-Only prints on flat surface
	-Nozzle moves based on pattern		-Larger particle size can block nozzle	-Printing vertical structure not possible
	-Non-contact printing, protects substrate from contamination or damage		-Low viscosity low volatility	-Small feature size can be achieved
Screen printing	-Stencil or mesh needed as mask	Moderate	-Highly viscous ink needed	-Can print on non-flat surface
	-A squeegee type device is used to force ink through the mesh		-No limitation about particle size	-Can print thick film
				-Small feature size cannot be achieved
Roll-to-roll printing	-Stencil or mesh needed as mask	Fast	-Medium to highly viscous ink needed	-Can print on flexible surface
	-A rotary structure needed to move the substrate		-No limitation about particle size	-Printing layers on top of each other is difficult due to alignment issues
	-Hard contact compression is used to transfer the ink to the substrate			
Aerosol jet printing	-Compressed air flow required	Slow	-Inks needs to be volatile	Can print on non-flat substrate
	-Non-contact printing, protects substrate from contamination or damage			
	-Larger nozzle size can tolerate larger ink particles			
Brush painting	-Stencil/mask needed	Slow	-High viscous ink needed	-Can print on non-flat substrate
	-Cheap method, brush needs to be moved manually ovar the stencil			-Small feature size cannot be achieved
				-Can print thick film

## Device and application

In this section, we present different structures of TEGs and their proposed application. The main design challenge in a TEG is to maximize the temperature difference across the junctions. Keeping this in mind, we have categorized TEGs into two groups: (1) planar TEGs where the junctions are on the same plane as the substrate and (2) through-plane TEGs where the junctions are in the cross-plane direction of the substrate. In most of the heat sources, like the human body or a hot pipe, the surface remains at the same temperature. So, to create a temperature difference across planar devices, researchers have proposed innovative device designs such as corrugation, origami, z-scheme folding and vertical staking.^103–106^ Here we focus on techniques that have been implemented using printing methods.

**Fig. 4:**
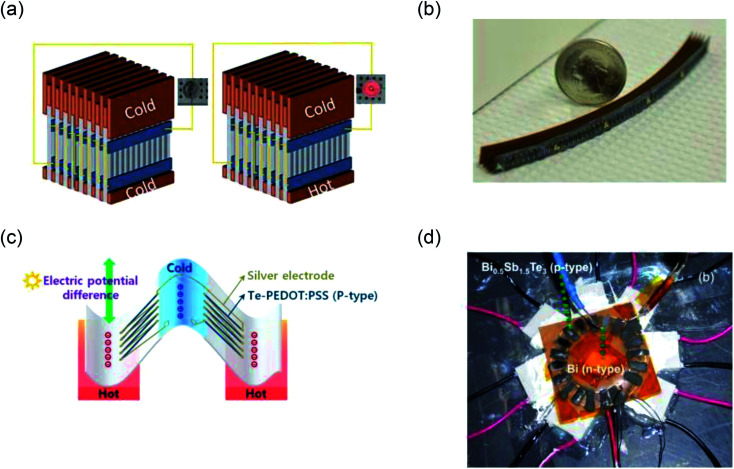
Device made from planar TEG (a) schematic illustration of planar TEG stacked vertically (adapted with permission form ref. 87). (b) Dispenser printed vertically stacked planar TEG on flexible substrate (adapted with permission from ref. 55). (c) Schematic illustration of the folded structure proposesd in ref. 91. (d) Radially oriented planar TEG (adapted with permission from ref. 52).

One solution to create temperature difference across planar device is to put the device vertically on the hot surface. Iezzi *et al.* placed planar TEGs vertically on a hot plate. The TEGs were able to power a RFduino and send temperature reading to a cell phone.^107^ Qingshuo *et al.* printed planar thermocouple on paper, then stacked up to 300 pieces of papers, and used them vertically between the hot and cold surface^87^ (Fig. 4(a)). The stacked papers were connected *via* copper sheet or wires. As the output voltage was low, a step up converter was used. For a 100 °C temperature difference, this device generated enough energy to power an LED. The group later refined their method using thermal lamination to achieve an output power of 24 μW cm^−2^ at 50 °C temperature difference.^108^ Wang *et al.* proposed a similar approach of stacking planar TEG.^57^ Using 250 thermocouples, they could generate 203.5 μW of power at 20 °C temperature difference. They used both the planes of the substrate (Fig. 4(b)). Thermoelectric elements were printed on the top layer, and connectors were printed on the bottom layer while through hole vias were used to connect between the layers. The group also printed micro-batteries and used the TEGs to charge them. Another reported approach is to organize the TE element radially, keeping the heat source at centre. Yuan *et al.* screen printed BiTe based TE legs radially and used a radio-isotope at the centre to create temperature difference.^109^ Madan *et al.* also proposed radially oriented planar TEGs, as shown in Fig. 4(d). A power density of 1230 μW cm^−2^ at 70 K was achieved from the radial TEG.^54^ Menon *et al.* used a mixture of radial arrangement and stacking.^110^ However, they could only reach 15 nW cm^−2^ at 45 K temperature difference mainly due to the low-efficiency material used. Another popular technique to utilize planar TEG is to coil them up.^78^ Cao *et al.* used their screen-printed TEG to coil up into a cylinder.^111^ Their output power was 12.7 nW cm^−2^ for 20 °C temperature difference. It should be noted that the inside of the cylinder was hollow, which reduced the output power density. Bending radius is an important factor for coiled up TEGs that will dictate how closely the thermocouples can be packed.^111^ Bae *et al.* proposed another novel structure of folding their TEGs, which are dispenser printed on flexible substrate as shown schematically in Fig. 4(c).^92^ They could achieve an output power of 1 nW cm^−2^ for a temperature difference of 10 °C (Table 7).

**Table 7:** Performance comparison of different orientation of planar TEGs

Orientation	Printing method	Material	Output (μW cm^−2^)	Ref.
Vertical stacking	Screen printing	PEDOT:PSS	24, Δ*T* = 50 K	108
Vertical stacking	Dispenser printing	Bi_2_Te_3_ and Sb_2_Te_3_		57
Radial arrangement	Dispenser printing	Bi and Bi_0.5_Sb_1.5_Te_3_	1230, Δ*T* = 70 K	54
Radial arrangement	Screen printing	BiTe		109
Radial arrangement + stacking	Brush painting	Polymer based	0.015, Δ*T* = 45 K	110
Coil up	Screen printing	BiTe/SbTe	0.012, Δ*T* = 20 K	111
Folded structure	Dispenser printing	Polymer-based	0.001, Δ*T* = 10 K	92

In through-plane TEGs thermoelectric elements are grown vertically to the plane of the substrate. They generally consist of two planes, where temperature difference can be applied. All the conventional TEGs are through-plane devices. However, printing through plane devices is challenging since most of the printing technique prints very thin films.

**Fig. 5:**
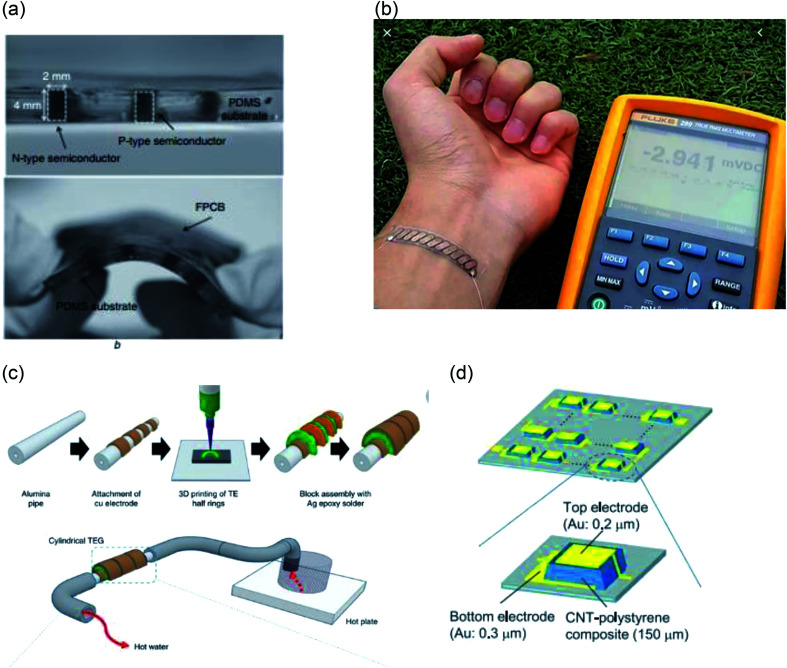
Through plane TEGs (a) through plane TEG embedded in PDMS substrate (adapted with permission from ref. 34). (b) Band-aid style through plane TEG (adapted with permission from ref. 86). (c) Schematic illustration of the process of 3-d printing TEG on hot pipe (adapted with permission from ref. 111). (d) Schematic illustration of screen printed CNT based TEG (adapted with permission from ref. 88).

Jo *et al.* used a PMMA mould to make holes in PDMS thick film and dispenser printed TE inks in the holes^34^ (Fig. 5(a)). The thickness of the device was 4 mm. PDMS was chosen due to its desireable properties of flexibility and electrically and thermally insulating behavior. Their device was able to generate 84 nW cm^−2^ at 19 °C temperature difference. Recently, Zhang *et al.* were able to print through-plane 3-D TEGs using roll to roll printing.^102^ Their device reached an output power density of 1.49 mW m^−2^ at 25 °C temperature difference. They also demonstrated an integrated body heat harvester that can power an LED. A coin-sized supercapacitor was used to boost the generated voltage. Kim *et al.* used extrusion based printing to 3-D print through-plane TEGs.^112^ The method was capable of printing TEGs on any geometries. They demonstrated a real-world application by 3-D printing half ring TEGs of an alumina pipe (Fig. 5(c)). The device achieved an output power density of 1.42 mW cm^−2^ at a temperature difference of 39 °C. Another innovative technique is to use fabric as the printing substrate. The ink filtrates through the fabric *via* capillary effect whilst the fabric acts as a support structure. So, no external substrate is needed. Kim *et al.* screen printed such TEGs on glass fabric.^86^ Their device reached 3.5 mW cm^−2^ for 50 °C temperature difference (Fig. 5(b)). Soumeri *et al.* used CNT–polystyrene composite to fabricate flexible and lightweight TEG as shown in Fig. 5(d) achieving output power of 55 mW m^−2^ at 70 °C temperature difference.^88^

## Conclusions

The field of thermoelectricity relies on optimizing the parameters that have opposing performance effects. Printing these thermoelectric materials involves further optimization of the ink and printing techniques. Researchers have investigated various thermoelectric materials, substrates and methods with the aim of increase the efficiency of the TEGs. Notwithstanding the excellent progress, there remains significant room to further improve performance and optimise inks, especially those based on organic materials. In terms of inorganic material, the key to improve performance will be to develop inks that require lower curing temperature. With the increasing demand for wearable electronics and the great promise for TEGs to power them, there is significant scope for improvements in this exciting research field.

## Conflicts of interest

There are no conflicts to declare.

## Supplementary Material
